# Genetic analysis toward more nutritious barley grains for a food secure world

**DOI:** 10.1186/s40529-022-00334-z

**Published:** 2022-03-10

**Authors:** Samar G. Thabet, Dalia Z. Alomari, Henrik Brinch-Pedersen, Ahmad M. Alqudah

**Affiliations:** 1 Department of Botany, Faculty of Science, Fayoum University, Fayoum, 63514 Egypt; 2 Department of Agroecology, Aarhus University, 4200 Flakkebjerg, Slagelse, Denmark

**Keywords:** Zinc, Iron, Selenium, Barley, Micronutrient, GWAS

## Abstract

**Background:**

Understanding the relationships between nutrition, human health and plant food source is among the highest priorities for public health. Therefore, enhancing the minerals content such as iron (Fe), zinc (Zn) and selenium (Se) in barley (*Hordeum vulgare* L.) grains is an urgent need to improve the nutritive value of barley grains in overcoming malnutrition and its potential consequencing. This study aimed to expedite biofortification of barley grains by elucidating the genetic basis of Zn, Fe, and Se accumulation in the grains, which will contribute to improved barley nutritional quality.

**Results:**

A genome-wide association study (GWAS) was conducted to detect the genetic architecture for grain Zn, Fe, and Se accumulations in 216 spring barley accessions across two years. All the accessions were genotyped by single nucleotide polymorphisms (SNPs) molecular markers. Mineral heritability values ranging from moderate to high were revealed in both environments. Remarkably, there was a high natural phenotypic variation for all micronutrient accumulation in the used population. High-LD SNP markers (222 SNPs) were detected to be associated with all micronutrients in barley grains across the two environments plus BLUEs. Three genomic regions were detected based on LD, which were identified for the most effective markers that had associations with more than one trait. The strongest SNP-trait associations were found to be physically located within genes that may be involved in grain Zn and Fe homeostasis. Two putative candidate genes were annotated as Basic helix loop helix (BHLH) family transcription factor and Squamosa promoter binding-like protein, respectively, and have been suggested as candidates for increased grain Zn, Fe, and Se accumulation.

**Conclusions:**

These findings shed a light on the genetic basis of Zn, Fe, and Se accumulation in barley grains and have the potential to assist plant breeders in selecting accessions with high micronutrient concentrations to enhance grain quality and, ultimately human health.

**Supplementary Information:**

The online version contains supplementary material available at 10.1186/s40529-022-00334-z.

## Background

Micronutrient deficiency, also referred as hidden hunger, is now one of the most serious threats to human health in the twenty-first century. More than 2 billion people worldwide are affected by Fe and Zn deficiency-related disorders (Welch and Graham 2004). Recently, a high prevalence of nutritional deficiency and malnutrition has been reported in the countries relying on cereal diet, and high-risk communities include women and children (Graham 2008; Pandey et al. 2016). Every year, 5 million children die as a result of micronutrients deficiency (Lancet 2007). Therefore, exploring the genetic architecture of grain micronutrients in barley helps in improving grain quality and its dietary value.

Zinc (Zn) is a vital cofactor for several enzymes and regulatory proteins and its deficiency causes immune system problems, taste perception, and retardation of growth (Kambe et al. 2014; Krishnappa et al. 2017). Many enzymes in plants that are involved in the metabolism of auxin and carbohydrates, as well as the synthesis of regulatory proteins, require zinc as a cofactor (Cakmak 2000; Rehman et al. 2018).

Iron (Fe) is an essential micronutrient for human health, and its insufficiency has negative consequences such as slowing physical growth and negatively impacting motoric development, resulting in fatigue and low growth (Bouis 2002, 2003). For plant, Fe is required for a variety of essential physiological and metabolic mechanisms (Briat et al. 2007; Morrissey and Guerinot 2009; Rout and Sahoo 2015), also it is considered an essential part of vital processes such as photosynthesis, respiration, and nitrogen fixation, as well as for participation in the electron transport chain and cytochrome. Therefore, Fe is suitable for agricultural production in both cultivated and natural species (Soetan et al. 2010; Tang et al. 1990). Therefore, improving Fe and Zn concentrations in plant crops has a significant impact on grain yield, nutrient intake, and human health by overcoming malnutrition and its associated problems (Alomari et al. 2018, 2019; Graham et al. 1999).

Another essential mineral element for humans is selenium (Se) (Haug et al. 2007; Kumssa et al. 2015). Since then, its active role as an antioxidant, anticancer, antibacterial, antiviral activity, and general immune function regulator has been highlighted, while its insufficiency has been related to a number of diseases, such as hypothyroidism and osteoarthritis (Rayman 2000). The majority of selenium nutrient intake is less than the daily requirement of 50–55 µg (Rayman 2000; Schwarz and Foltz 1957). Around 0.5–1 billion people worldwide do not consume sufficient Se and are at risk of several diseases (Haug et al. 2007; Kumssa et al. 2015).

Genetic biofortification of important crops, such as wheat and barley is the most feasible plant breeding strategy, which used to alleviate micronutrient deficiency and develop mineral-rich crop varieties that are beneficial to humans health (Rawat et al. 2013; Singh et al. 2016).

Due to the complex genetic nature of micronutrient accumulation in barley grains, a genome-wide association study (GWAS) as an effective tool was applied for identifying the genetic factors controlling such complex inherited traits (Alomari et al. 2021, 2017; Alqudah et al. 2020; Hamblin et al. 2011). Several QTLs, including Zn and Fe, were found to be significantly associated with grain micronutrients in different plant crops, such as wheat, (Srinivasa et al. 2014; Tiwari et al. 2009; Velu et al. 2017; Xu et al. 2012), barley (Hussain et al. 2015), and rice (Pradhan et al. 2020). Recently, hot spot QTLs of Se has been reported in lentils (Ates et al. 2016) and rice (Norton et al. 2010; Zhang et al. 2010). Five QTLs were detected to be significantly associated with grain Se micronutrient accumulation in wheat (Pu et al. 2014). Therefore, it is an imperative to study the genetic factors controlling micronutrient concentrations, such as Zn, Fe, and Se in barley grains, as well as to validate and apply these findings in marker-assisted selection.

The current investigation aims to mine genomic regions/candidate genes underlying the natural phenotypic variation of micronutrient grain concentrations, such as Zn, Fe, and Se in 216 worldwide spring barley accessions during two field growing seasons (2019/2020 and 2020/2021). Here, we detected 222 SNPs associated with the natural variation of Zn, Fe, and Se elements using association genetics. Putative candidate genes responsible for mineral accumulation in barley grains were reported. The annotation of candidate genes showed their pivotal roles in improving grain and food quality that ultimately human health.

## Material and methods

### Plant material and field trials

A set core collection of 218 worldwide spring barley accessions was obtained from the genebank, IPK-Gatersleben, Germany. Two field trials were conducted at the Experimental Station of the University of Fayoum during two seasons (2019/2020 and 2020/2021). The trials were designed using a randomized complete block design with four replicates. Barley grains were planted directly into clay loam soil and were grown in plots of three 1 m^2^ of each accession spaced by two rows within each plot. The field plots were subjected to standard local agronomic practices. After harvest, fifty kernels of each accession were randomly selected to measure thousand-kernel weights (TKW) with a digital weighing balance and were used to prepare the grains for milling. After that, the milled barley grains were dried in an oven overnight at 40 °C.

### Determination of micronutrient accumulation

Micronutrient concentrations (Zn, Fe, and Se) were determined according to the method of Zulfiqar et al. (2020). Samples of barley grain flour were taken to be digested by di-acid (HClO_4_:HNO_3_ at 3:10 v/v ratio) mixture and placed on a digestion plate (Heidolph, USA model, MR3003). Afterward, the atomic absorption spectrophotometer (Shimadzu, UV-1201, Kyoto, Japan) was used to determine Zn, Fe, and Se concentrations in grain samples.

### Statistical analysis

The broad-sense heritability (*H*^2^) was calculated using the following equation:

VG/[VG + (Ge/nE)].where VG is the genotype variance, Ge is the residual variance and nE is the year number.

Analysis of variance (ANOVA) was calculated for each mineral across the years, and significant differences between genotypes and years were detected at a probability level of p ≤ 0.05. Pearson’s correlation coefficient was used to assess the relationships among the measured parameters at a p value of 0.05 (Wei and Simko 2017). Using the lme4 package, Residual Maximum Likelihood (REML) was used to analyze the phenotypic data and the Best Linear Unbiased Estimates (BLUEs) were used to measure the phenotypic means of all micronutrient concentrations in barley (Bates et al. 2015).

### Genome-wide association scan for the studied traits

The barley population was genotyped with SNP molecular markers from next generation sequences. The physical positions of SNPs were defined according to Morex genome sequence v2 (Monat et al. 2019). In the population, around 8 K SNPs are distributed over the whole genome (Milner et al. 2019). GWAS analysis was performed using the FarmCPU model in the GAPIT R package (Lipka et al. 2012), which evaluated the BLUE values for all the studied elements. The FarmCPU model, which was used for the GWAS analysis, as an effective method for detecting and controlling false-positive associations (Liu et al. 2016). The procedure of GWAS analysis and validation is described by Alqudah et al. (2020).

### Candidate gene underlying the studied traits

Significant markers that located inside the linkage disequilibrium (LD) were used to mine the potential candidate genes. High-confidence (HC) candidate genes were identified using Morex v2 (Monat et al. 2019) and the BARLEX database https://apex.ipk-gatersleben.de/apex/f?p=284:10.

Furthermore, candidate genes expression profiles were measured as FPKM (fragments per kilobase of transcript per million mapped reads) using the RNA-seq expression database. Only grain-related organs were explored in gene expression profiling research, including CAR5, 15: caryopses at 5 and 15 DPA (days post-anthesis), LEM: Lemma [6 weeks PA (post-anthesis)], LOD: Lodicule (6 weeks PA), PAL: Palea (6 weeks PA), EPI: Epidermis (4 weeks), RAC: Rachis (5 weeks PA). This approach has been recently reported by Alqudah et al. (2021).

## Results

### Description of phenotypic data

**Fig. 1:**
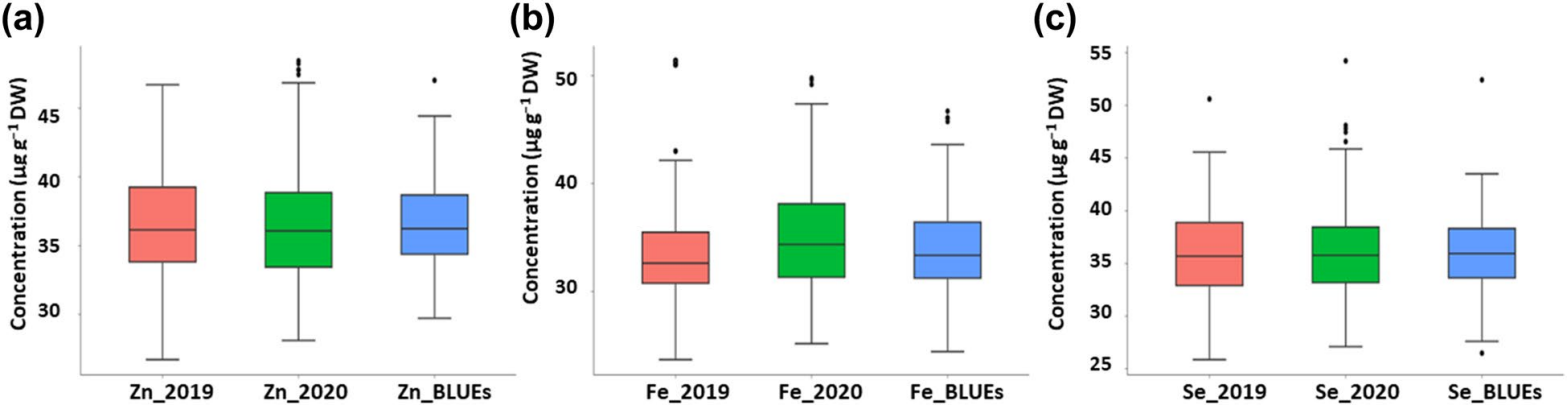
Box plots of genotypes for each year (2019/2020) and BLUEs in spring barley; **a** Zn concentration, **b** Fe concentration and **c** Se concentration

**Fig. 2:**
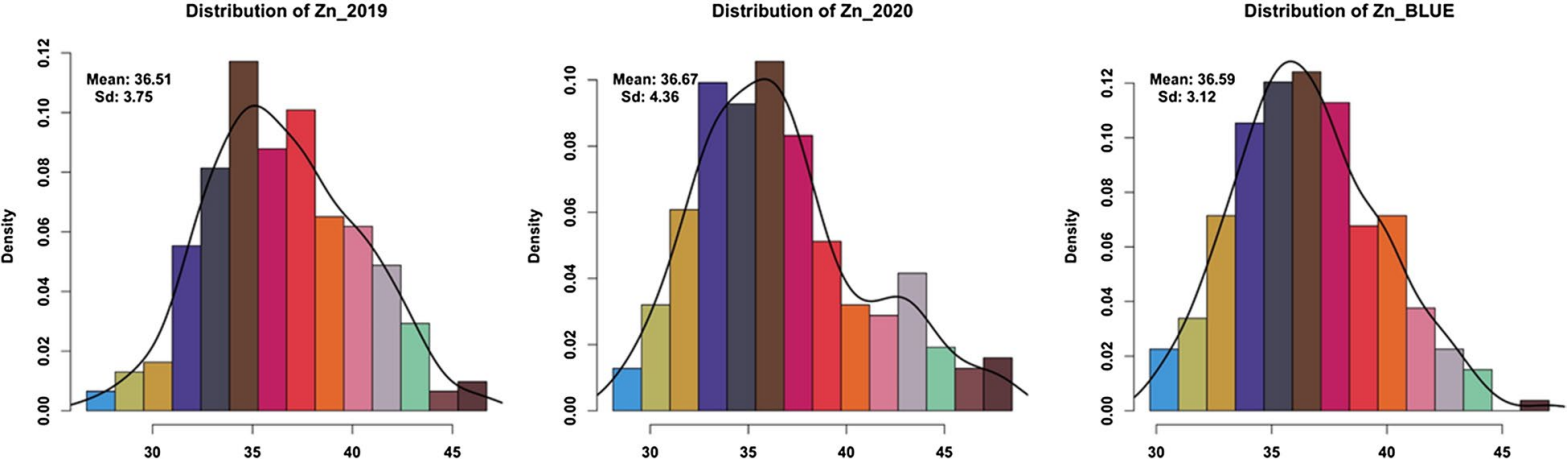
Distribution of phenotypic data in all genotypes for Zn concentration for each year (2019/2020) and BLUEs in spring barley

**Fig. 3:**
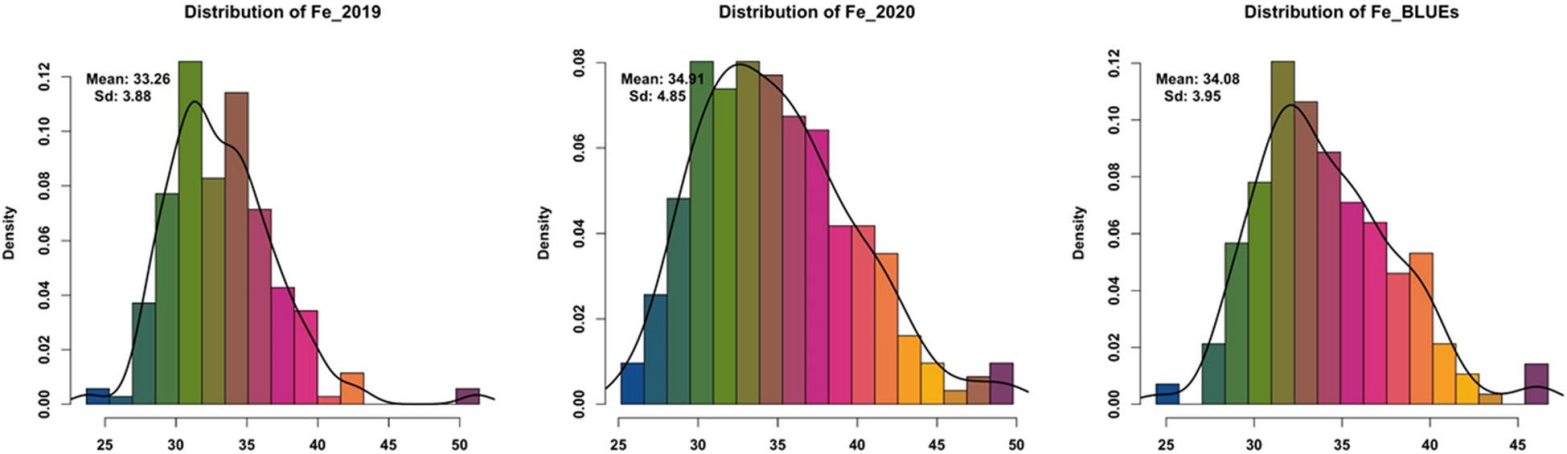
Distribution of phenotypic data in all genotypes for Fe concentration for each year (2019/2020) and BLUEs in spring barley

**Fig. 4:**
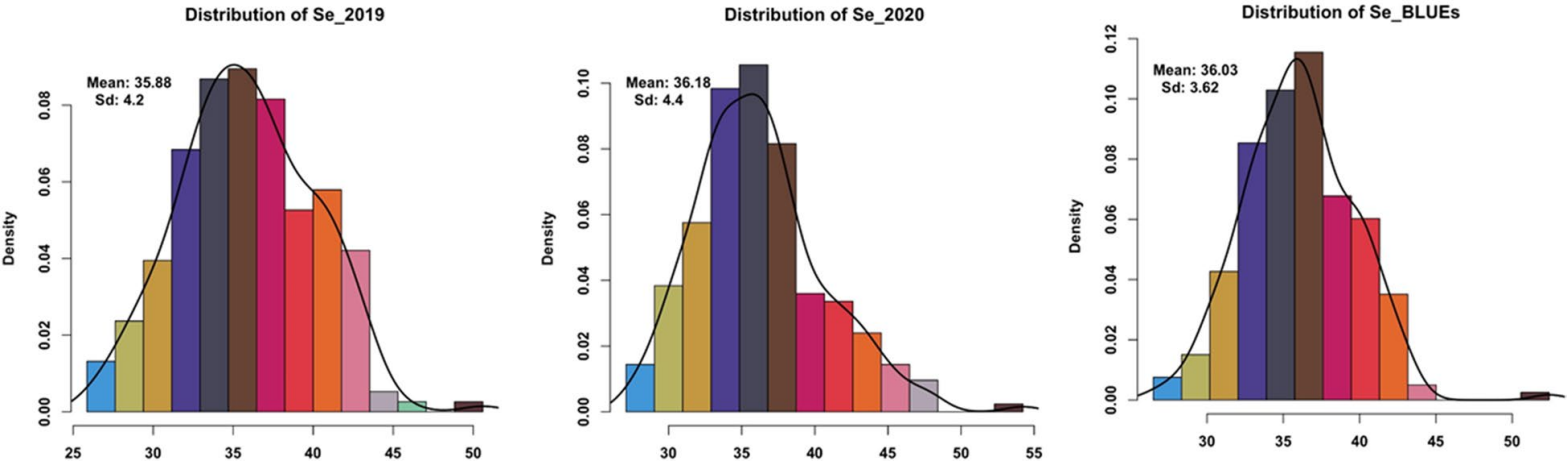
Distribution of phenotypic data in all genotypes for Se concentration for each year (2019/2020) and BLUEs in spring barley

For all traits analyzed, significant differences were found in the population during both environments plus BLUEs due to G × E interactions (Additional file 1: Table S1). High natural phenotypic variations was detected for Zn, Fe, and Se micronutrient accumulation across two seasons (Fig. 1, Additional file 1: Table S2). Normal distribution for each trait measured in all barley accessions was detected (Figs. 2, 3, 4). In the season 2019, the highest measured grain Zn, Fe, and Se accumulation were 46.71, 51.38, and 50.60 µg g^−1^ DW, respectively while in 2020 the highest Zn, Fe, and Se values were around 48.45, 49.78, and 54.23 µg g^−1^ DW, respectively (Additional file 1: Table S1). The estimated BLUEs for Zn, Fe, and Se were 38.37, 35.56, and 39.45 µg g^−1^ DW, respectively (Additional file 1: Table S1).

Markedly, Fe showed high heritability values of 75.65% for both environments, revealing that phenotypic values were relatively stable for all accessions over the 2 years (Additional file 1: Table S2). However, Zn and Se showed moderate heritability (30.81% and 58.52%), whereby a lot of the phenotypic variance was represented by the year and error variance components.

**Fig. 5:**
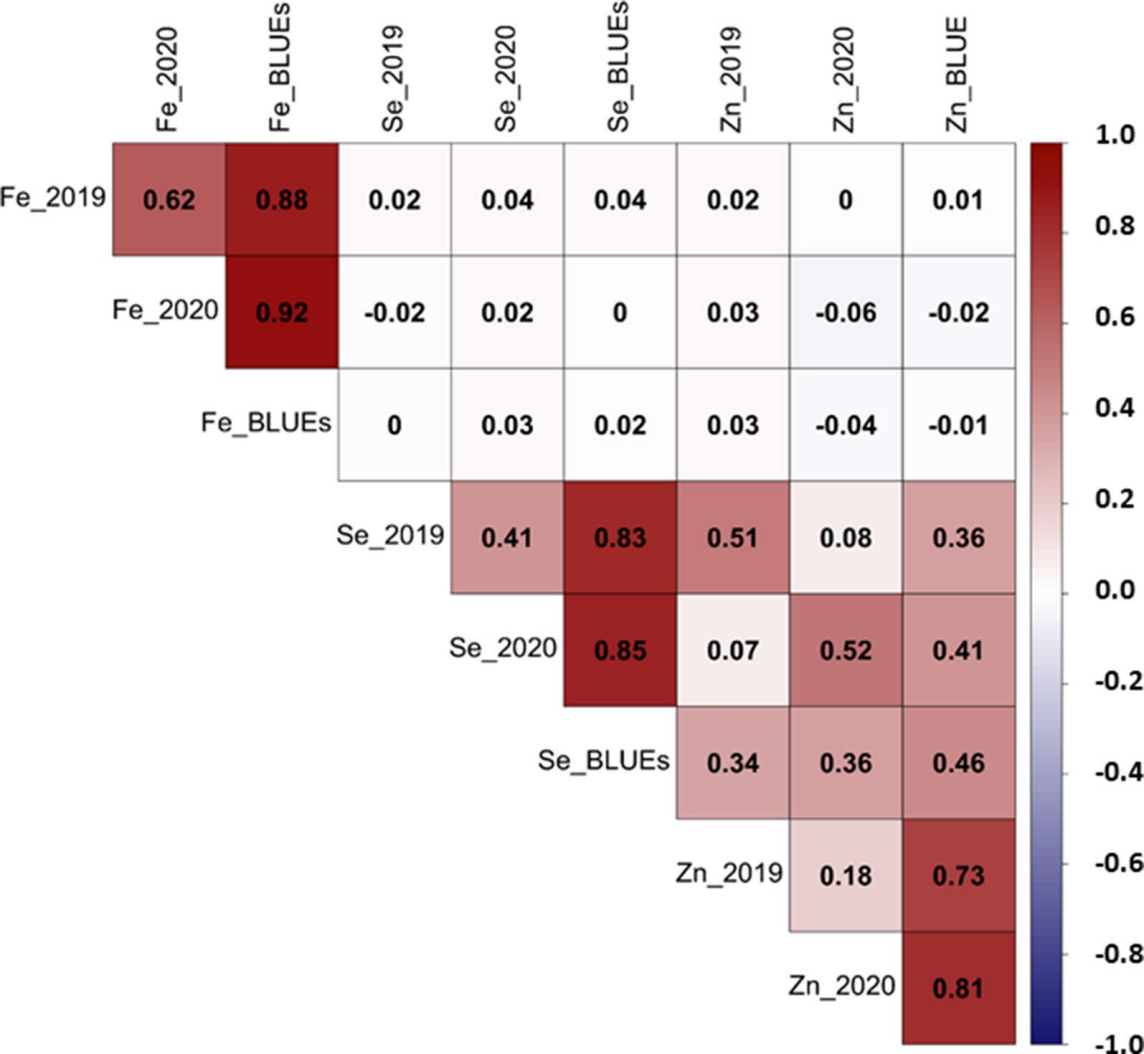
Correlations of all the studied traits including Zn, Fe and Se for each year (2019/2020) and BLUEs in spring barley

The Pearson’s correlation measured for all mineral traits among the two environments and BLUEs is presented in Fig. 5. The highest correlation was found between Fe_BLUEs and Fe_2019 and Fe_2020 (r = 0.88*** and 0.92***), respectively. Positive correlation was significantly obtained between Zn_BLUEs and Zn_2019 and Zn_2020 (r = 0.73*** and 0.81***), respectively. Moreover, highly positive correlation was observed between Se_BLUEs and Se_2019 and Se_2020 (r = 0.83*** and 0.85***), respectively. On the other hand, moderate positive correlation was seen between Zn_BLUEs and Se_2019, Se_2020 and Se_BLUEs (r = 0.36**, 0.41** and 0.46**), respectively. Interestingly, a negative correlation was found between Zn_BLUEs and Fe_2020 and Fe_BLUEs (r = − 0.02 and -0.01), respectively. The phenotypic correlation indicates that Zn and Se could share genomic regions (Fig. 5).

### Genetic analysis and genes underlying minerals in Barley

**Fig. 6:**
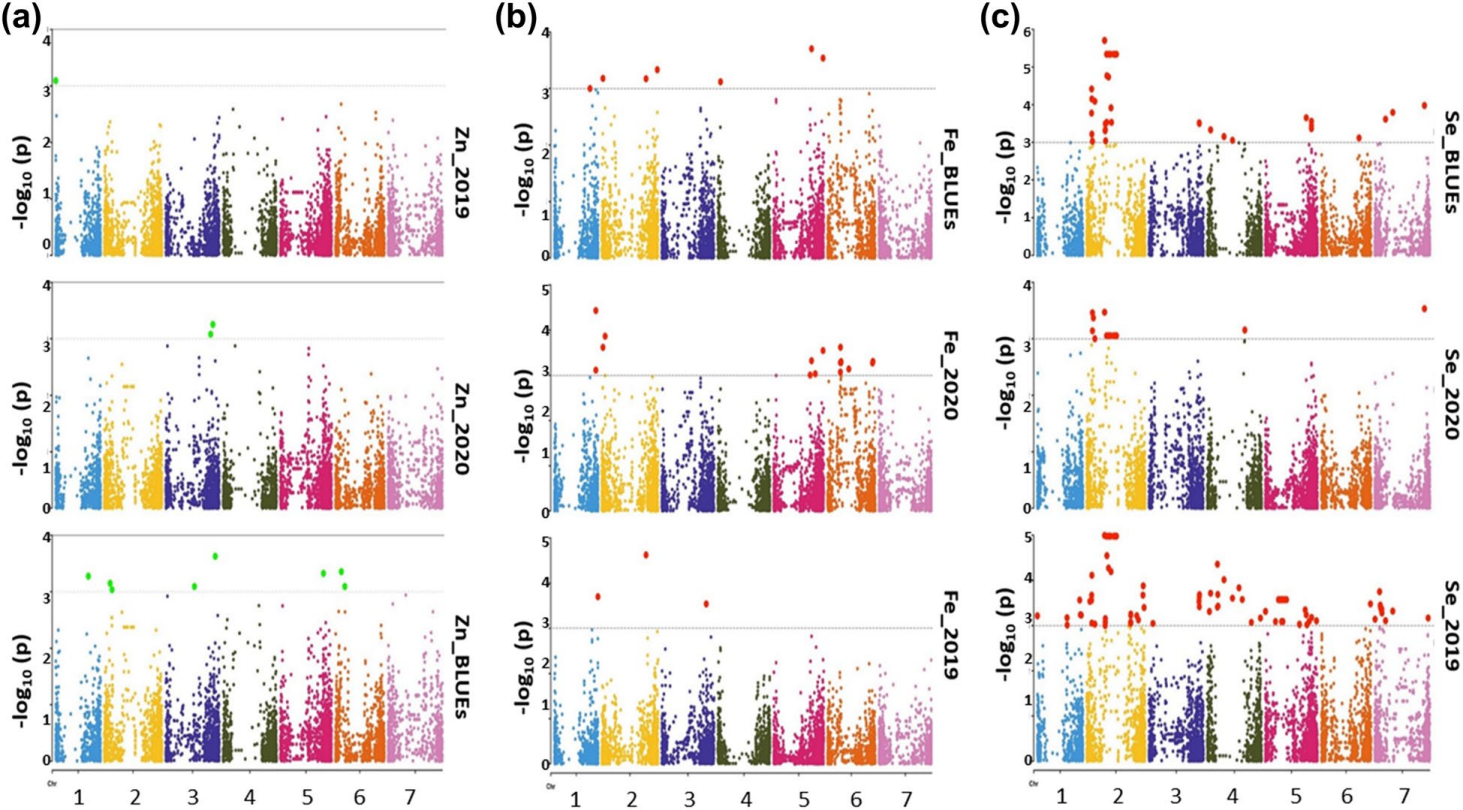
Summary of genome-wide association scans of Zn (**A**), Fe (**B**), and Se (**C**) for all barley genotypes (216) which were genotyped with a high-density 9 K SNPs array from Illumina™ for each year (2019/2020) and BLUEs. The horizontal red color line indicated the threshold of − log_10_ (p value) of 3

Multi-locus GWAS identified 222 significant [− Log_10_ (p) ≥ 3.0] associations that harbors all micronutrients for each year in addition to BLUEs (Fig. 6, Additional file 1: Table S3). These SNPs were located over seven chromosomes, with 89 on chromosome 2H, followed by 5H (36 SNPs), 4H and 7H (21 SNPs each), 1H and 6H (20 SNPs each), 3H (15 SNPs), (Fig. 6, Additional file 1: Table S3).

Interestingly, 167 SNP markers were significantly detected for Se accumulation, followed by Fe (42 SNPs) and Zn (13 SNPs), which were present in both seasons and BLUEs (Fig. 6).

Exclusively, three genomic regions were discovered based on LD and located on chromosomes 3H, 5H, and 7H. For instance, on chromosome 3H, BOPA1_6402-691 SNP (578,601,859 bp) was associated with Zn_BLUEs, Se_2019, and Se_BLUEs by three SNPs. In addition, BOPA1_2251-643 SNP at 7H (206,751,899 bp) was associated with Se and Zn_BLUEs by two SNPs (Fig. 6, Additional file 1: Table S5).

**Fig. 7:**
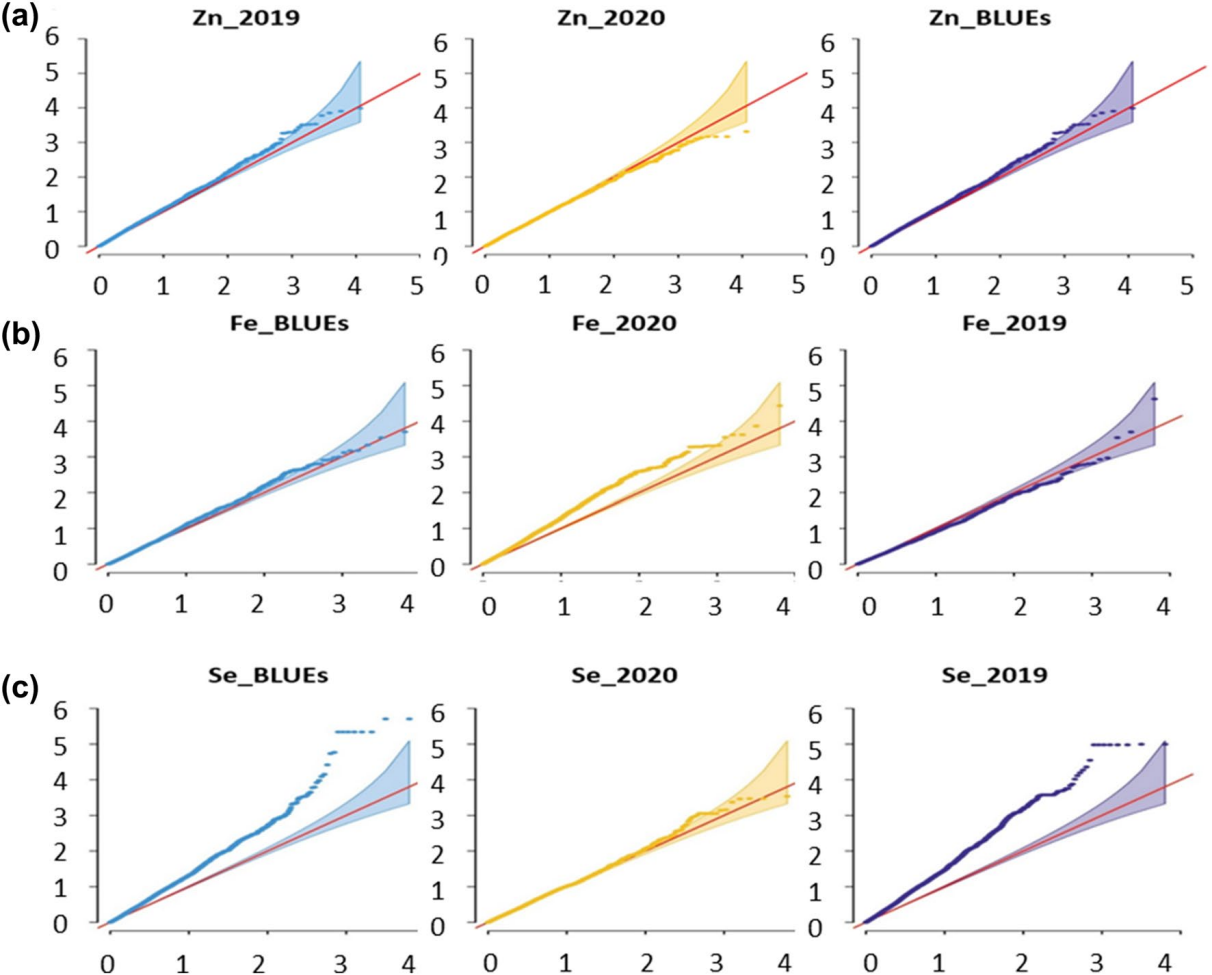
Summary of quantile–quantile scale representing expected versus observed − log_10_ (p value) of Zn (**A**), Fe (**B**), and Se (**C**) for all barley genotypes

The QQ plots demonstrated that the GWAS for most of the traits can be used for further analyses as there are little number of markers spreaded out of the confidance interval while the model was strong enough to control the markers with almost no over corrected markers. It leads to conclude that the observed association p value distribution was consistent with the expected association distribution (Fig. 7).

The gene content of the three genomic regions on chromosomes 3H, 5H and 7H harbors 394 potential candidate genes that were found to be highly associated with Zn, Fe, and Se among the growing environments and BLUEs (Additional file 1: Table S6). The first region is located on chromosome 3H and harbors two important candidate genes; *HORVU.MOREX.r2.3HG0258450* that annotated as Selenium-binding protein (SBP) at (578,710,262–578,713,489 bp) and *HORVU.MOREX.r2.3HG0258460* that encodes 2-oxoglutarate (2OG) and Fe(II)-dependent oxygenase superfamily protein (2-ODDs) at (578,715,293–578,716,369 bp). The second important region located on chromosome 5H harbors four candidate genes that regulated the variation of all the studied minerals including Zn, Fe, and Se in barley grains. For instance, *HORVU.MOREX.r2.5HG0411320* is the most well-known versatile gene, was mapped as An ATP-dependent zinc metalloprotease, FtsH at (500,037,722–500,044,141 bp). Interestingly, on chromosome 7H (205,216,091–205,221,133 bp), the Squamosa promoter binding-like protein (SPL) gene family is detected that enhanced barley Zn and Se micronutrients (Table 1).

**Table 1:** The list of candidate genes based on the linkage disequilibrium of multi-traits associated marker

Genomic region	Gene	Chr	Start	End	Gene length	Annotation
1	*HORVU.MOREX.r2.3HG0258450*	3	578,710,262	578,713,489	3228	Selenium-binding protein
1	*HORVU.MOREX.r2.3HG0258460*	3	578,715,293	578,716,369	1077	2-oxoglutarate (2OG) and Fe(II)-dependent oxygenase superfamily protein
2	*HORVU.MOREX.r2.5HG0411320*	5	500,037,722	500,044,141	6420	ATP-dependent zinc metalloprotease FtsH
2	*HORVU.MOREX.r2.5HG0411850*	5	501,094,176	501,094,607	432	Protein FAR1-RELATED SEQUENCE 5
2	*HORVU.MOREX.r2.5HG0412340*	5	502,454,312	502,455,148	837	Basic helix loop helix (BHLH) family transcription factor
2	*HORVU.MOREX.r2.5HG0413150*	5	505,319,272	505,325,405	6134	Homeobox leucine zipper protein
3	*HORVU.MOREX.r2.7HG0567820*	7	205,216,091	205,221,133	5043	Squamosa promoter binding-like protein

**Fig. 8:**
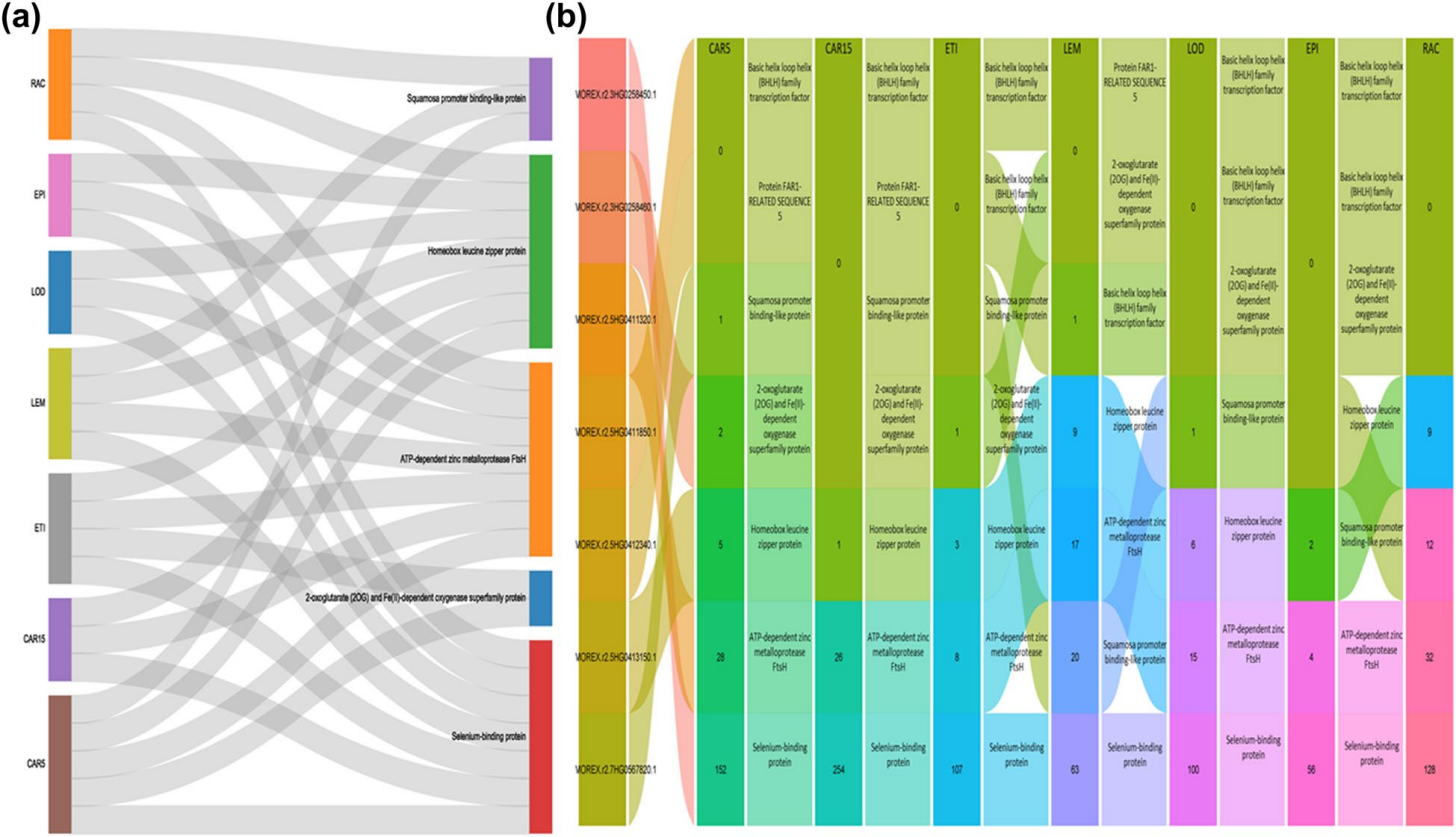
Expression pattern of the candidate genes in different barley grain related-organs at different developmental stages. The connection of the candiate genes with the grain organs showed in **a** while the expression level and pattern has been shown as alluvial plot in **b**

Interestingly, most of these genes, such as *HORVU.MOREX.﻿r2.7HG056782﻿0* and *HORVU.MOREX.r2.5HG0413150* are also expressed in grain-related organs such as CAR 5, 15 DPA, LEM, LOD, PAL, EPI, and RAC (Fig. 8). The differential expression reveal that such candidate genes act important biological specific role in grain filling and cell growth. The candidate genes were varied in expression within the grain organs whereas *HORVU.MOREX.r2.3HG0258450.1*, *HORVU.MOREX.r2.5HG0411320.1* and *HORVU.MOREX.r2.5HG0413150.1* genes were expressed in all tested grain-related organs (Fig. 8). Notably, *HORVU.MOREX.r2.3HG0258450.1*, annotated as Selenium-binding protein is the most overexpressed gene in all grain-related organs, particularly in CAR5 and CAR15.

## Discussion

Breeding of essential micronutrients in barley is a potential approach for implementing efficient routine selection in barley breeding programs. Understanding the natural genetic variation in 216 spring barley accessions is therefore important for mining the genomic regions and definening the potential candidate genes that contribute to improve mineral accumulation in barley grains. A wide range of natural variation was detected for the mineral concentrations including Zn, Fe, and Se based on BLUEs that equaled 38.37, 35.56, and 39.45 µg g^−1^ DW, respectively. Our findings agreed with the report by Herzig et al. (2019) for grain minerals in spring barley. High heritability was detected for Fe concentration by 75.65% across the two environments. This is attributed to genotypic effects, which is consistent with previous research (Garcia-Oliveira et al. 2009; Peleg et al. 2009; Vreugdenhil et al. 2004). However, Zn and Se showed moderate heritability (30.81% and 58.52%), indicating that the phenotypic variance was due to the year and error variance. A similar range of heritability values was found by Herzig et al. (2019) for grain minerals in barley NAM population. A significant positive correlation was found between Zn and Se concentrations, indicating the presence of common genetic factors controlling the accumulation of both minerals. Hence, such outputs provide evidence to develop a cost-effective strategy to improve nutritional traits in barley breeding programs.

Therefore, our study offers high nutritive accession which can be used to improve the nutritional quality of barley-based food products as flour or for improving grain quality through breeding high nutritional quality of new varieties. These are important ways to promote human health and dietary improvement using barley-based products foods.

### Candidate genes

Candidate genes were identified for the most effective markers that had associations with more than one trait. Based on GWAS analysis, 222 significant SNPs were detected for grain Zn, Fe, and Se accumulation, that were mapped across all chromosomes and clustered into significant genomic regions based on LD. Exclusively, three genomic regions harboring 394 potential candidate genes were discovered on chromosomes 3H, 5H, and 7H that were found to be highly associated with Zn, Fe, and Se among the growing environments and BLUEs. The strong potential candidate gene at 3H is *HORVU.MOREX.r2.3HG0258450* that annotated as Selenium-binding protein (SBP) and highly expressed during grain development and in grain related-organs demonstrating that this gene plays role in Se accumilation and other minerals (Zhao and Castonguay 2015).

*HORVU.MOREX.r2.3HG0258460* candidate gene that encodes 2-oxoglutarate (2OG) and Fe(II)-dependent oxygenase superfamily protein (2-ODDs), act as a co-factor for iron and found to be involved in the oxidative reactions of the plant metabolic pathways (Farrow and Facchini 2014). The expression of this gene in grain related-organs was very low that does not fit with the hypothesis of playing a role in mineral accumulations in the barley grain.

The second important genomic region located on chromosome 5H harbors four candidate genes that regulated the variation of all the studied minerals including Zn, Fe, and Se in barley grains. The most prominent multifunctional gene is *HORVU.MOREX.r2.5HG0411320* at (500,037,722–500,044,141 bp) annotated as an ATP-dependent zinc metalloprotease, FtsH is the major thylakoid membrane protease required for photosynthetic pathways in plants (Kato and Sakamoto 2018). Substantial proportions of the micronutrient metals (i.e., Cu, Fe, Mn, and Zn) are assigned to proteins required for plant photosynthetic process, indicating their significance in plant-specific biochemistry (Yruela 2013). Markedly, this gene was relatively highly expressed in all tested grain organs suggesting its crucial role in mineral accumulation in barley grains that needs further molecular validation.

In the same genomic region, two candidate genes are coding transcription factors; *HORVU.MOREX.r2.5HG0411850* that encodes Protein *FAR1-RELATED SEQUENCE 5* and *HORVU.MOREX.r2.5HG0412340* that encodes a Basic helix loop helix (BHLH) transcription factor. Both FAR1 and BHLH were shown to explain the variation of Zn, Fe, and Se, implying that they attributed to improving grain mineral accumulation. *FAR-RED ELONGATED HYPOCOTYLS3* (*FHY3*) and its homolog. Recent studies have demonstrated that FHY3/FAR1 are key regulators in a wide range of many metabolic, developmental and physiological processes in different plant species during photoperiod (Li et al. 2011). Together, FHY3/FAR1 play important role in the development of chloroplasts and the chlorophyll biosynthetic pathway during early seedling development (Tang et al. 2012).

The bHLH transcription factor FER, a regulator of iron uptake responses in the root, was identified in the tomato mutant (Ling et al. 2002). This gene is known as one of the most key regulators of Fe homeostasis, which modulates Fe levels in *Arabidopsis* (Tanabe et al. 2019). Recently, wheat studies revealed that bHLH was found in all *TaMTP* promoter regions, and plays a critical role in metal homeostasis, which indicates its involvement in the Zn accumulation in grain cereals (Menguer et al. 2018; Vatansever et al. 2017). Even though their expression in the tested organs was not high compared with other candidates, we still believe our GWAS-based findings declared the potentiality of this cluster of genes in conferring mineral accumulation in barley. Moreover they are pleiotropic genes thus; a selection for the region harboring them can improve many traits at once.

A homeobox-leucine zipper protein *HOX4* that is annotated as *HORVU.MOREX.r2.5HG0413150* and control the variation of all the studied minerals (i.e., Zn, Fe, and Se). Similar results were found by Alomari et al. (2018) who reported that *TaHDZIP1 *is a candidate gene that has been linked to Zn concentrations in wheat grain. Several *TabZIP *genes may have a significant role in ion transportation in wheat (Li et al. 2015). A subset of *TabZIP* genes (Inaba et al. 2015) were shown to be upregulated under Zn deficiency. This gene was moderately expressed in all grain related-organs that suggests its importance in grain development and mineral accumulations.

Interestingly, on chromosome 7H (205,216,091–205,221,133 bp), the candidate gene from SPL gene family was detected, which is coding for transcription factor. This gene plays vital roles in plant development and grain-related traits (Birkenbihl et al. 2005; Klein et al. 1996). In the current study *SPL* genes controlled the variation of Zn and Se content, this agrees with other studies in *Arabidopsis*, *SPL* genes have a siginficant role in the regulation of transition metal homeostasis including Cu and Zn (Schulten et al. 2019). In rice, expression analysis of *OsSPL13* positively enhanced the regulation of cell size in the grain hull, resulting in improved grain-related traits, especially grain length (Si et al. 2016). Jiao et al. (2010) demonstrated that overexpression of *OsSPL14* has been shown to improve shoot and panicle branching, resulting in greater grain yield and performance. Furthermore, Wang et al. (2012) demonstrated that *OsSPL16 *is involved in cell division and grain filling, as well as all grain-related traits. Interestingly, Thabet et al. (2021) identified many candidate genes that play significant roles in plant growth, and seed germination in response to salt stress, of which *HvSPL6* was reported for the first time in barley. The expression of the *HvSPL* candidate gene in this study was detected during caryopses and lemma development suggested the important functions of *HvSPL* genes in micronutrient accumulation, particularly Zn and Se in different grain-related organs.

## Conclusion

The current study uncovers the genetic factors that control the natural variation in grain Zn, Fe, and Se accumulation in barley’s grain, providing basis for targeted plant breeding programs towards major crop cereals with improved micronutrient levels. Three genomic regions harbor putative candidate genes such as *HOX4* and *SPL*, that have been suggested as candidates for increasing grain Zn, Fe, and Se accumulation. Such outputs provide evidences that grain quality can be improved through exploiting the genetic variation that in turn improve food barley-based and promote human health.

## Supplementary Information

**Additional file 1: Table S1.** Phenotypic data of grain micronutrient concentration values among 2 years (2019/2020) and BLUEs for 216 barley genotypes. **Table S2.** Analysis of variance (ANOVA) and heritability of Micronutrient concentration in 216 barley accessions among two environments. **Table S3.** Marker trait associated with the studied traits. The physical position of markers which are passing -log_10_ (p value) of 3. **Table S4.** Marker trait associated with the multi traits. The physical position of markers which are passing -log_10_ (p value) of 3. **Table S5.** The list of genomic regions based on the linkage disequilibrium of multi-traits associated marker. **Table S6.** The list of candidate genes based on the linkage disequilibrium of multi-traits associated marker.

## Data Availability

All data generated during the study are interpreted in the manuscript.

## Abbreviations

Zn: Zinc
Fe: Iron
Se: Selenium
BLUEs: Best linear unbiased estimates
GWAS: Genome-Wide Association Study
FarmCPU: Fixed and random model Circulating Probability Unification
